# Case Image: Cutaneous Leiomyoma

**DOI:** 10.1002/ccr3.70471

**Published:** 2025-04-23

**Authors:** Sunil Jaiswal, Shraddha Uprety, Pratichya Thapa, Prakriti Lamichhane

**Affiliations:** ^1^ Department of Dermatology Chitwan Medical College Bharatpur Nepal

**Keywords:** clustered papules, cutaneous leiomyoma, histopathological diagnosis, smooth muscle

## Abstract

Cutaneous leiomyomas are rare benign smooth muscle tumors that often present as painful dermal nodules; however, they can be asymptomatic too. Histopathology remains the gold standard for diagnosis and warrants screening for Hereditary Leiomyomatosis and Renal Cell Carcinoma (HLRCC) in patients presenting with multiple lesions.

A 66‐year‐old female presented with multiple asymptomatic brown papules on the right flank region of the back arranged in a clustered pattern for the last 5 years, as shown in image 1. The lesions were gradually increasing in number. A punch biopsy of one of the papules was done and the histopathological images under magnification 10× and 40× are depicted in images 2 and 3, respectively.

## 1 What is the diagnosis?

- Neurofibroma
- Dermatofibroma
- Cutaneous Leiomyoma
- Lichen Planus
- Cutaneous Mastocytosis

## 2 Discussion

**FIGURE 1:**
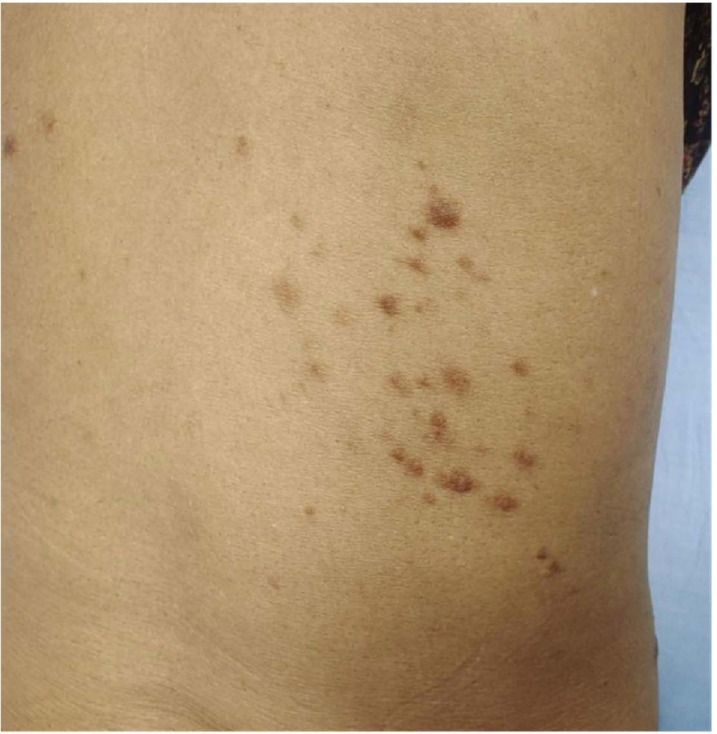
Multiple brown clustered papules on the trunk.

**FIGURE 2:**
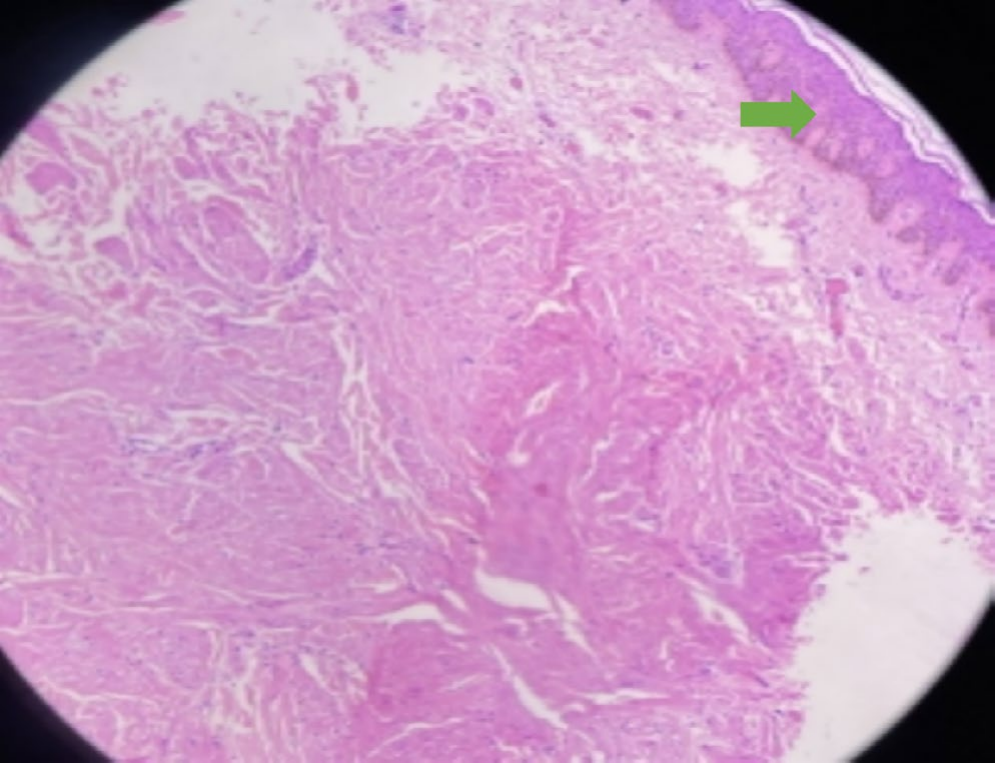
Hematoxylin and eosin‐stained section shows epidermis lined by keratinized stratified squamous epithelium with acanthosis (*10×, Green arrow).

**FIGURE 3:**
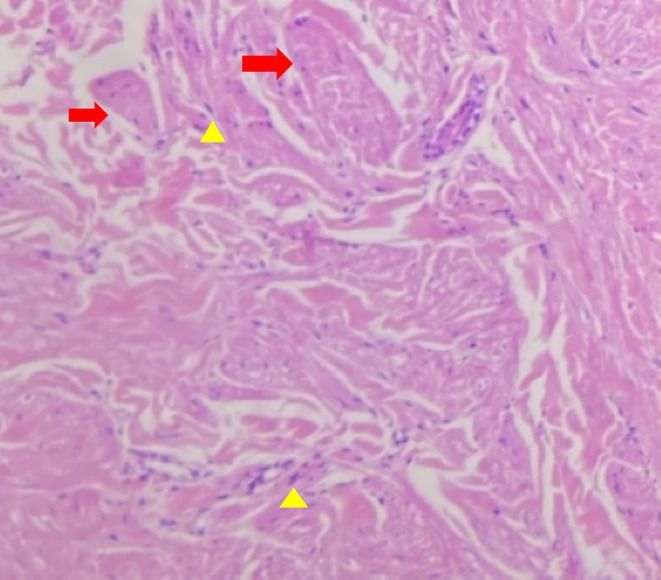
Dermis shows a circumscribed area of fascicles and interlacing smooth muscle bundles (Red arrows). Individual smooth muscle cells have elongated, thin nuclei with blunt ends and with pink cytoplasm (*40×, Yellow arrowheads).

Cutaneous manifestations in Figure 1 show multiple brown papules arranged in a clustered pattern on the trunk. The differential diagnosis considered was neurofibroma, dermatofibroma, cutaneous leiomyoma, cutaneous mastocytosis, and lichen planus. Hematoxylin and eosin‐stained section shows epidermis lined by keratinized stratified squamous epithelium with acanthosis. The underlying dermis shows a circumscribed area of fascicles and interlacing smooth muscle bundles. Individual smooth muscle cells have elongated, thin nuclei with blunt ends, cigar‐shaped, with pink, eosinophilic cytoplasm, Figures 2 and 3. Thus, the diagnosis is cutaneous leiomyoma. The patient was advised for genetic testing and planned for laser ablation.

Cutaneous leiomyoma is a rare benign smooth muscle tumor. It can originate at the arrector pilorum muscles of the hair follicle, dartos of the scrotum, areola, muscles of the vulva, and vascular smooth muscles. Based on the origin, cutaneous leiomyomas can be classified into piloleiomyomas, angioleiomyomas, and genital leiomyomas. It may be sporadic or inherited. Multiple cutaneous leiomyomas often occur due to heterozygous germline mutation in the fumarate hydratase enzyme gene [1]. Cutaneous leiomyomas occur singly; however, multiple lesions can be present. They may occur in association with uterine leiomyomas and renal cell carcinoma in the hereditary leiomyomatosis renal cell cancer (HLRCC) syndrome [2]. Patients usually present with nodules ranging in size from 2 mm to 20 mm and may be skin, pink, red, or red‐brown. Lesions might be painful in patients with multiple leiomyomas. Histological examination reveals interweaving bundles of smooth muscle cells with characteristic “blunt‐ended cigar‐shaped nuclei” and eosinophilic cytoplasm [1]. The gold standard therapy for solitary cutaneous leiomyoma is surgical excision; however, recurrence is a common problem. Treatment options for multiple cutaneous leiomyomas include cryosurgery, carbon dioxide laser, and pharmacological therapy (nifedipine, nitroglycerin, and doxazosin). Gabapentin, pregabalin, duloxetine, and injection of botulinum toxin can be given for symptomatic relief [1]. Patients developing solitary lesion in a sporadic pattern usually have an excellent prognosis after surgical excision in comparison to patients with multiple and inherited forms [3]. A regular follow‐up is necessary for patients with multiple cutaneous leiomyomas for early detection of any internal malignancy [2].

## Author Contributions

**Sunil Jaiswal:** conceptualization, writing – original draft, writing – review and editing. **Shraddha Uprety:** conceptualization, investigation, writing – original draft. **Pratichya Thapa:** conceptualization, formal analysis, methodology. **Prakriti Lamichhane:** conceptualization, methodology, supervision.

## Ethics Statement

The patient in this manuscript has given written informed consent for the use of their case details (including photographs) for publication.

## Conflicts of Interest

The authors declare no conflicts of interest.

## Data Availability

The data that support the findings of this study are openly available in repository name, (doi), reference number (reference number).
